# Age-Related Differences in the Gastrointestinal Microbiota of Chinstrap Penguins (*Pygoscelis antarctica*)

**DOI:** 10.1371/journal.pone.0153215

**Published:** 2016-04-07

**Authors:** Andrés Barbosa, Vanessa Balagué, Francisco Valera, Ana Martínez, Jesús Benzal, Miguel Motas, Julia I. Diaz, Alex Mira, Carlos Pedrós-Alió

**Affiliations:** 1 Departamento de Ecología Evolutiva, Museo Nacional de Ciencias Naturales, CSIC, Madrid, Spain; 2 Departament de Biologia Marina i Oceanografia, Institut de Ciències del Mar, CSIC, Barcelona, Spain; 3 Departamento de Ecología Funcional y Evolutiva, Estación Experimental de Zonas Áridas, CSIC, Almería, Spain; 4 Instituto de Investigaciones en Materiales, Universidad Nacional Autónoma de México, México DF, México; 5 Facultad de Veterinaria, Universidad de Murcia, Murcia, Spain; 6 Centro de Estudios Parasitológicos y de Vectores, CCT La Plata (CONICET-UNLP), La Plata, Argentina; 7 Department of Genomics and Health, FISABIO Foundation, Center for Advanced Research in Public Health, Valencia, Spain; Institute of Ecology, GERMANY

## Abstract

The gastrointestinal tract microbiota is known to play very important roles in the well being of animals. It is a complex community composed by hundreds of microbial species interacting closely among them and with their host, that is, a microbial ecosystem. The development of high throughput sequencing techniques allows studying the diversity of such communities in a realistic way and considerable work has been carried out in mammals and some birds such as chickens. Wild birds have received less attention and in particular, in the case of penguins, only a few individuals of five species have been examined with molecular techniques. We collected cloacal samples from Chinstrap penguins in the Vapour Col rookery in Deception Island, Antarctica, and carried out pyrosequencing of the V1-V3 region of the 16S rDNA in samples from 53 individuals, 27 adults and 26 chicks. This provided the first description of the Chinstrap penguin gastrointestinal tract microbiota and the most extensive in any penguin species. Firmicutes, Bacteoridetes, Proteobacteria, Fusobacteria, Actinobacteria, and Tenericutes were the main components. There were large differences between chicks and adults. The former had more Firmicutes and the latter more Bacteroidetes and Proteobacteria. In addition, adults had richer and more diverse bacterial communities than chicks. These differences were also observed between parents and their offspring. On the other hand, nests explained differences in bacterial communities only among chicks. We suggest that environmental factors have a higher importance than genetic factors in the microbiota composition of chicks. The results also showed surprisingly large differences in community composition with other Antarctic penguins including the congeneric Adélie and Gentoo penguins.

## Introduction

The gastrointestinal tract (GIT) can be considered an ecosystem in which host cells and bacteria interact [1]. Such interactions affect the main function of GIT, that is, the digestion and absorption of food intake by individuals, which has an influence on other physiological functions. The intestinal microbiota may also protect hosts from pathogens [2]. Therefore, it is very relevant to determine the composition of the GIT microbiota. Information about the composition of the gastrointestinal microbiota in animals is not homogeneous. Despite considerable available information about mammals, studies on birds have been mainly carried out on poultry (e.g. [3]) and the literature about the microbiota of wild birds is scarce (see reviews in [2,4–5]).

In general, studies on avian gastrointestinal microbiota have been done with adult individuals. Thus, there are very few studies describing the microbiota of chicks and the changes between young and adult birds [6–11]. Although some microbes may enter the eggs before hatching, the GIT is colonized by bacteria from different sources shortly after [12]. The inoculum of bacteria after hatching comes from the nest environment [13], through the food delivered by the parents [14] or by contact with conspecifics [15]. Moreover, several factors such as diet or climatic variables (temperature and humidity) also influence the composition of GIT microbiota in birds [16,7].

The microbiota of Antarctic seabirds and particularly those of Antarctic penguins are among the less studied in spite of an early interest in determining the presence of bacteria in their GIT [17,18]. Although there are some studies dealing with specific aspects of gastrointestinal microbiota such as human transmission ([19], see also [20] for a review, and references therein), information about the whole community of gastrointestinal bacteria of some penguin species together with other Antarctic seabirds has been published only recently for Adélie [21] and for King, Macaroni, Gentoo, and Little penguins [22].

The aim of the present work is to describe for the first time the gastrointestinal microbiota in the Chinstrap penguin (*Pygoscelis antarctica*) comparing both adults and chicks and to analyze the likely differences between them. In this study we focus on cloacal microbiota because it reflects very closely the assemblages present in the GIT [23] and does not require invasive sampling.

## Materials and Methods

### Sampling

The study was conducted in the Vapour Col penguin rookery in Deception Island (63° 00’S 60° 40’W) in December 2008 and January 2009. Permission to work in the study area and for penguin handling was granted by the Spanish Polar Committee (http://www.idi.mineco.gob.es/portal/site/MICINN/menuitem.7eeac5cd345b4f34f09dfd1001432ea0/?vgnextoid=9b6fefb8b7c0f210VgnVCM1000001d04140aRCRD&lang_choosen=en). Forty nests with two eggs were selected during the incubation phase and marked with wood sticks. Nests were visited every two days to control for hatching date. Penguins were captured in nests by hand, and samples were taken from both adults and both nestlings in 35 nests. During adult sampling, chicks were kept in a bag to avoid heat loss or predation. Cloacal samples were taken using swabs when chicks were seven days old. Swabs were kept in sterilized tubes. After sampling, chicks were replaced in the nest and adults were released close to nest. All the adults immediately resumed care of the chicks. Swabs were frozen during 10–15 days until DNA extraction was carried out. DNA could not be retrieved from several samples and, thus, the final number of samples used was 27 adults and 26 chicks from 18 nests. Nests with two chick and two adult samples numbered 14.

### DNA extraction

Nucleic acids from the whole microbial community were extracted using the QIAamp DNA Stool Mini Kit (Qiagen, Germany), following manufacturer’s instructions. Samples were quantified and quality checked with NanoDrop ND-1000 spectrophotometer (Thermo Scientific, USA) and the nucleic acid extracts were stored at -20°C.

### Pyrosequencing and data analysis

An aliquot of 20 ng μl^-1^ of DNA extract was prepared for amplification and subsequent pyrosequencing at the Research & Testing Laboratory (Texas, USA; http://www.researchandtesting.com). Eubacterial primers 28F (5’-GAGTTTGATCNTGGCTCAG-3’) and 519R (5’-GTNTTACNGCGGCKGCTG-3’) were used for amplification of the V1-V3 region of the16S rDNA gene (Bacterial 16S Assay b.2, annealing temperature 54°C) and pyrosequencing was carried out in a Roche 454 GS FLX Titanium system.

Reads were analyzed using QIIME 1.6 ([24]; http://qiime.org/) except for the chimera detection, which was run in mothur v.1.33.1 ([25]; http://www.mothur.org/). Reads between 125–600 bp long were used for analysis. They were first checked for quality (sliding window Phred average 50bp>25) and then, sequencing noise was removed using denoiser ([26]). Chimera detection was done with ChimeraSlayer ([27]) based on the alignment file released by SILVA 108 database (http://www.arb-silva.de). Sequences were clustered into Operational Taxonomic Units (OTUs) using the UCLUST method ([28]), with a 97% threshold of similarity and including the “—optimal option.” A representative sequence (the most abundant one) for each OTU was picked for further taxonomic assignment with BLAST classifier, using sequence similarity from a file of reference sequences provided by SILVA 115 database (http://www.arb-silva.de). Based on these results, an OTU table was initially constructed and then filtered in order to remove Archaea, chloroplasts, and previously detected chimeras, thus producing the final OTU table. Sequences have been deposited in the metagenomics MG-RAST public database (http://metagenomics.anl.gov/) with ID number 4666568.3.

### Analysis of diversity

The OTU table was used for constructing a matrix that was subsampled to the same number of total reads per sample and SquareRoot transformed. Based on this, a Bray-Curtis distance matrix was produced and used to plot the Kruskal’s nonmetric multi-dimensional scaling ordination (NMDS). Differences between the two categories (age and nest) were tested with two-way ANOSIM (Analysis Of SIMilarity; [29]). NMDS, ANOSIM, and SIMPER were calculated with PRIMER (Plymouth Routines in Multivariate Ecological Research, version 6, UK). The similitude between GIT microbiota of chics and their parents was analyzed by means of a GLM (Generalized Linear Model) with Bray-Curtis distance values as the dependent variable and adult kinship (parents/non-related adults) as independent factor. Nest was included as a random factor. Chao and Shannon indexes were calculated using R [30] and the Vegan package [31], after random sampling of the same number of reads.

The rarefacted matrix was used for statistical analysis. Rarefaction was carried out randomly subsampling all the samples to the same number of total reads in two ways. 1) down to 3591 reads when using all the samples (53) and 2) down to 9413 reads when excluding the samples that had less than 9 000 reads (33_P_113, 37_A_81, 27_P_18, 16_P_126, 33_A_98), leaving the number of samples at 48.

## Results

### Description of the data set

**Fig 1:**
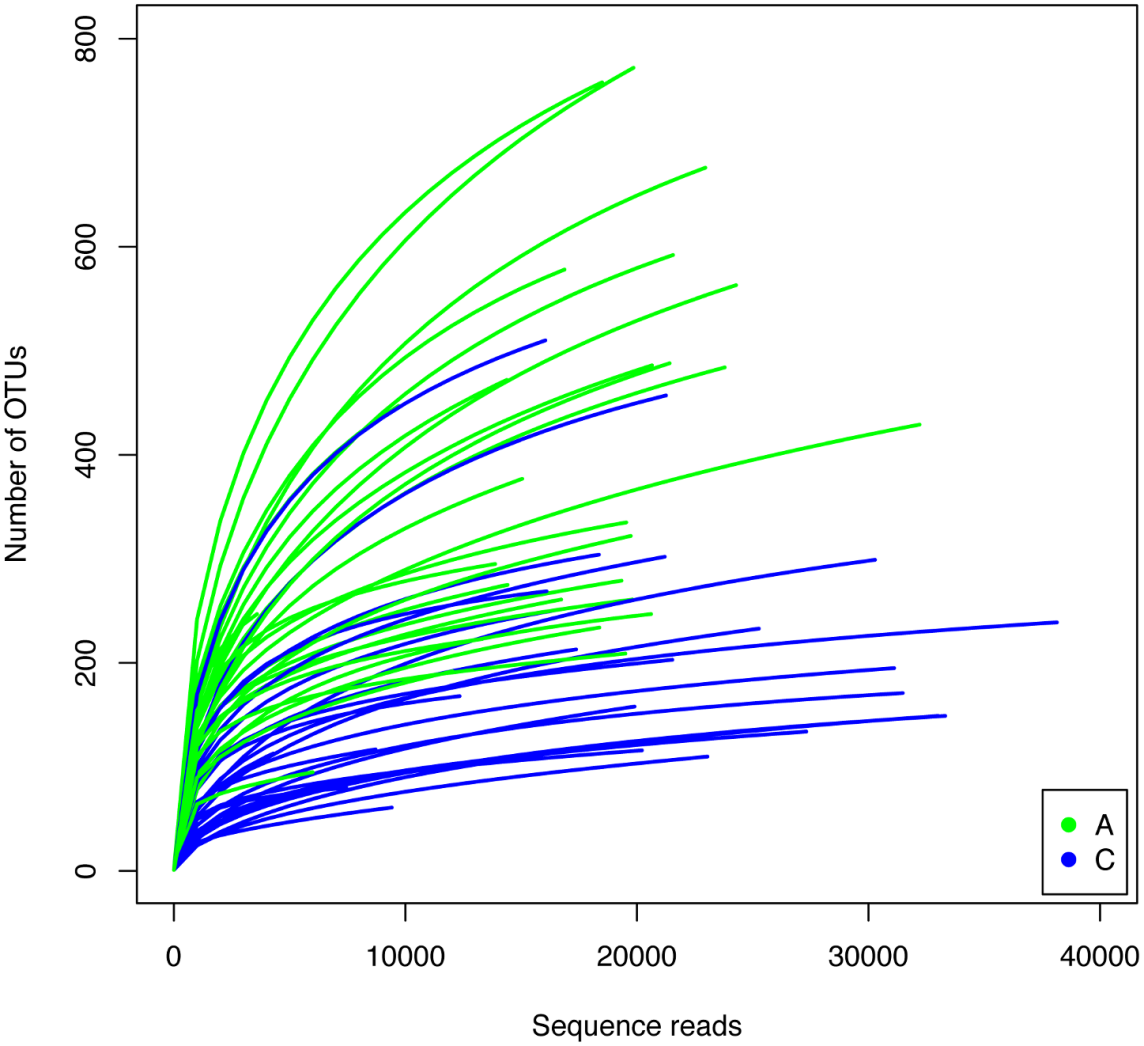
Taxa accumulation curves for all samples: adults in green and chicks in blue. For comparisons samples were rarefied down to 9,413 reads and samples with less than 9,000 reads were not used. In general, curves for chicks were closer to saturation than those of adults.

**Fig 2:**
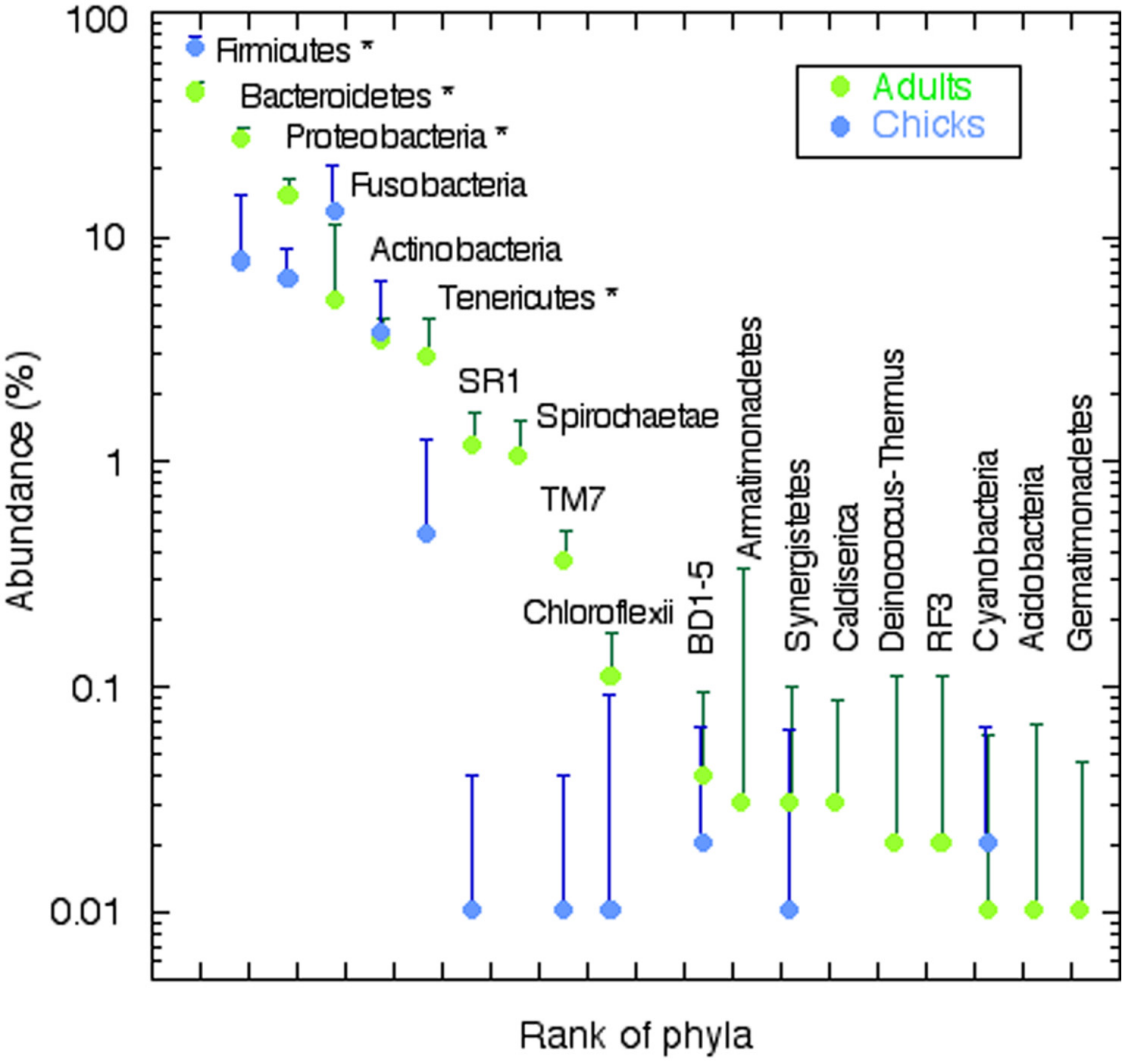
Rank-abundance curves for adults (green symbols) and chicks (blue symbols) considering taxonomy at the phylum level. An asterisk indicates the phyla whose abundance was significantly different between adults and chicks. The ten most abundant phyla are shown with horizontal labeling. Those with vertical labeling belong to the rare biosphere (less than 0.1% of the reads) and only a selection of the phyla in Table 2 is shown. Vertical lines show 95% confidence intervals (only upper ones shown for clarity). Dots for abundance of Armatimonadetes, Caldiserica, Deinococcus, RF3, Acidobacteria and Gematimonadetes in chicks do not appear because these were absent from the chick microbiota.

Over one million clean reads were obtained from 53 individuals, 27 adults and 26 chicks from 18 nests (Table 1). On average, each sample was represented by close to 19000 reads. When clustered at the 97% similarity level, 3621 OTUs were found in the whole data set. On average, the number of OTUs per sample was 298 with a relatively low number of singletons (73 on average). The number of OTUs was significantly lower in chicks than in adults (p<0.0001, two tailed t-test). In accordance with this, species accumulation curves for chicks showed a higher degree of saturation than those for adults (Fig 1). The number of singletons, another indication of how well the richness of the sample has been covered, was significantly lower for chicks. After rarefaction to 9413 reads (five samples had to be discarded because they had less than this number of reads) the number of OTUs per sample ranged between 332 for adults and 166 for chicks (Table 1). The corresponding Chao 1 estimators were 461 and 225 for adults and chicks respectively. The Shannon diversity index was also higher for adults than for chicks (Table 1). In summary, the number of OTUs was relatively low in all cases compared to marine [32,33,34] or soil samples [35,36], but similar to human gut microbiota [37], and the adults had a richer and more diverse microbiota, while the chicks showed higher dominance and, therefore, lower evenness. Such differences in dominance were apparent even at the phylum level (Fig 2). Adults and chicks shared the same main phyla of bacteria: Firmicutes, Bacteroidetes, Proteobacteria, Fusobacteria, Actinobacteria and Tenericutes. However, chicks showed a very marked dominance by Firmicutes and lower representation of Bacteroidetes and Proteobacteria than adults (Table 2). Adults had significantly more sequences of 13 phyla, while chicks had more abundance of only five.

**Table 1:** Number of individuals and nests sampled, number of reads retrieved, number of OTUs and singletons, Chao and Shannon’s indices for adults, chicks, and the whole data set.

Variable	Total	Adults	Chicks
**Individuals**	53	27	26
**Nests**	18	18	16
**Reads**^a^	1,006,428	478,065	528,363
**Average reads** ^b^	18,989 (1,049)	17,706 (1,144)	20,322 (1,790)
**Average OTUs** ^b^	298 (25)	393 (35)	200 (22)
**Average singletons** ^b^	73 (6)	96 (10)	48 (4)
**Average OTUs** ^c^	252 (20)	332 (25)	166 (19)
**Average Chao** ^c^	348 (29)	461 (39)	225 (25)
**Average Shannon** ^c^	2.97 (0.15)	3.75 (0.12)	2.16 (0.16)

^a^ Out of 1,298,696 raw reads.

^b^ Average per sample with standard error between parentheses.

^c^ Same as above after normalization.

**Table 2:** Number of reads of all the phyla in the data base (left) and of the classes of the most abundant phyla (right). The “Diff” columns indicate whether abundance was significantly higher in adults (A), in chicks (C), or not significant (blank) with a two tailed t test at p<0.001.

Phylum	Reads	Diff	Phylum	Class	Reads	Diff
Firmicutes	581632	C	Firmicutes	Clostridia	516128	C
Bacteroidetes	166240	A		Bacilli	42526	C
Proteobacteria	98273	A		Erysipelotrichia	13192	A
Fusobacteria	93985	C		Negativicutes	9786	A
Actinobacteria	35204	C				
Tenericutes	16642	A				
Candidate division SR1	6012	A	Bacteroidetes	Bacteroidia	139704	A
Spirochaetae	4495	A		Flavobacteria	16004	A
Candidate division TM7	1729	A		Sphingobacteriia	9846	A
Chloroflexi	689	A		Vadin HA17	386	A
Candidate division BD1-5	266	A		Cytophagia	245	
Synergistetes	206	A		SB-1	55	A
Cyanobacteria	148					
Caldiserica	140	A	Fusobacteria	Fusobacteriia	93985	C
Deinococcus-Thermus	124	A				
Candidate division RF3	109	A	Proteobacteria	Betaproteobacteria	39132	A
Acidobacteria	97			Gammaproteobacteria	38929	A
Armatimonadetes	62	A		Epsilonproteobacteria	16745	C
Candidate division BRC1	54	C		Alphaproteobacteria	1931	
Verrucomicrobia	40			Deltaproteobacteria	1534	A
Gemmatimonadetes	34					
Lentisphaerae	29		Actinobacteria	Actinobacteria	32037	C
Planctomycetes	26			Coriobacteriia	2748	A
Candidate division OP11	16			Acidimicrobiia	309	
Candidate division OD1	15	C		Thermoleophilia	61	C
Chlorobi	14			MB-A2-108	48	C
Candidate division WS3	1					
Deferribacteres	1		Other		31097	A
Fibrobacteres	1					
Total	1006428					

The complete taxonomic list included 21 phyla and eight candidate divisions (Table 2). Overall, Firmicutes was the most represented phylum. Classes Clostridia and Bacilli were the most abundant in chicks, while classes Erysipelotrichia, and Negativicutes were more abundant in adults. The next phylum was Bacteroidetes with Bacteroideia as the most abundant class. All classes were more abundant in adults, except Cytophagia. Beta- and Gammaproteobacteria were the most abundant Proteobacteria in adults, while Epsilonproteobacteria were the most abundant in chicks. Alphaproteobacteria were minor components of the microbiota. Fusobacteria had a similar abundance to total Proteobacteria and were significantly more abundant in chicks. Finally, Actinobacteria were also important, especially the class Actinobacteria in chicks, while class Coriobacteriia was more abundant in adults. Thus, differences between adults and chicks were not only found at the phylum level, but also their microbiotas were dominated by different classes from the same phyla.

**Fig 3:**
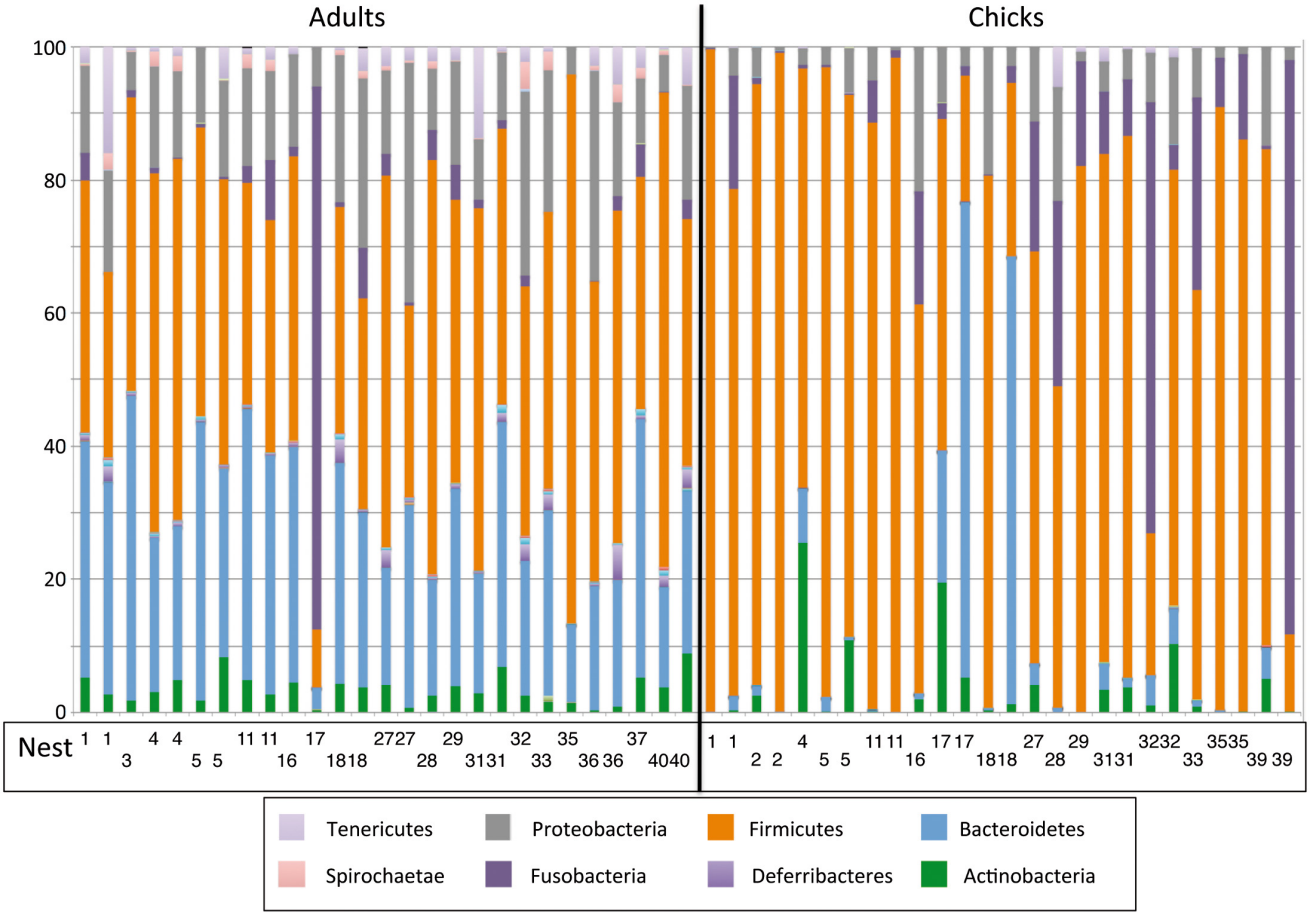
Taxonomic composition of the microbiota at the phylum level, for all the sampled individuals. The vertical black line separates adults from chicks. The nest of each individual is shown in the lower part. The color code is shown only for the main groups discernible in the graph.

The relative abundance at the phylum level for each individual is shown in Fig 3. Most adults showed similar compositions but a few individuals were clearly different. For example, the adult from nest 17 showed dominance by Fusobacteria and very limited representation of Bacteroidetes. The same was true for chicks, although in this case there was higher heterogeneity. For example, the second chick from nest 39 showed dominance by Fusobacteria and very few Firmicutes. These differences did not show any apparent relation with nest. The most abundant genera have been listed in S1 Table.

### Adults versus chicks

**Fig 4:**
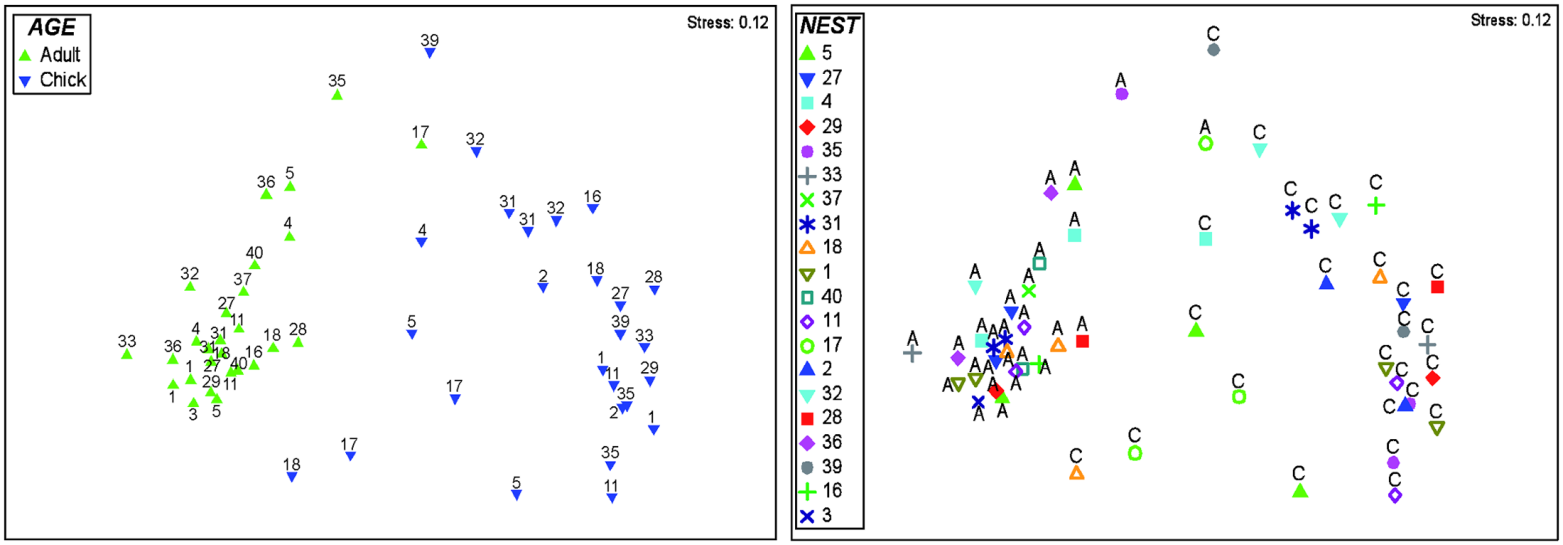
Non-metric multidimensional scaling (NMDS) diagram of all the samples. A: samples have been color coded by age, adults in green and chicks in blue. Numbers indicate nest. B: samples color coded by nest. A and C indicate age.

Bray-Curtis distances were calculated for all sample pairs and the results were used to construct an NMDS diagram (Fig 4). We have color coded the samples in two different ways: according to age (Fig 4a) and according to nest (Fig 4b) to facilitate visualization. Clearly, age was a more important variable than nest for the composition of the microbiota (Fig 4a). The NMDS separated adults from chicks along the horizontal axis. It was also apparent that microbiotas from adults were more similar among themselves than chick microbiotas, which showed a larger range of distance values.

On the other hand, no apparent pattern could be seen when samples were coded according to nest (Fig 4b). In some cases (such as nest 1, inverted empty green triangles) adults were very different from chicks, but the two adults and the two chicks were very close to each other. In other cases (nest 18, empty orange triangles) adults were close together while chicks were very different. Finally, in some cases (nest 5, green triangles) both adults and chicks were fairly different from each other. Chicks and adults from the same nest were never close.

These patterns were statistically analyzed by ANOSIM. A two-way nested analysis was carried out considering age and nest as the two factors. Age explained differences in bacterial communities after control for nests (R = 0.87, p = 0.002). However, nest did not explain variation after correcting by age (R statistic was -0.53, p = 1.000). The results were the same when only samples with more than 9000 reads were analyzed. When chicks and adults were analyzed separately, the nest explained differences among chicks (R = 0.30, p = 0.023), but not those of adults (R = 0.15, p = 0.168).

We wanted to check whether the microbiota of every chick was closer to that of its parents than to those of other adults. Since differences between all chicks and adults analyzed together were very large, it was possible that smaller effects such as this one were masked. In order to clarify this, we carried out a GLM taking the variable of similarities as the dependent factor, but including only comparisons among chicks and adults. The predictor factor was whether the comparison corresponded to chick-parent or to a non-related pair of individuals (but always adult-chick). Results of the similitude between chicks and their parents did not show significant differences when compared to the similitude between chicks and other (non parent) adults (F (1, 13) = 0.13, p = 0.72). However, we found differences in the similitude among nests: a few nests showed a high similitude between chicks and their parents but most nests did not show any similitude (F (13, 13) = 7.67, p = 0). Finally, the interaction between kinship (parent/non-related adults) and nest was not significant (F (13, 16) = 0.47 p = 0.90).

We carried out a SIMPER (SIMilarity of PERcentages) analysis to identify the OTUs with larger contributions to the differences between chicks and adults. Twenty OTUs accounted for 20.5% of the dissimilarity, with 12 being more abundant in adults and eight in chicks (S2 Table). The OTUs that were more abundant in chicks were mostly Clostridiales plus one *Leuconostoc* and one *Fusobacterium*. Those more abundant in adults, in turn, were mostly Bacteroidetes and Clostridiales, plus two Neisseriales and one each of Fusobacteriales, Campylobacterales and candidate div. SR1. All these OTUs were among the most abundant ones in the whole data set. Thus, differences among chicks and adults were due to the most abundant members of the GIT microbiota and not to differences in the rare ones.

## Discussion

The composition of the GIT microbiota of some species of penguins was known from a few studies. Banks et al. [21] constructed clone libraries with samples from several Adélie penguins. These authors sequenced 183 clones and clustered them into 52 OTUs at 99% similarity (Table 3). It is surprising that so few OTUs were found with such a high level of similarity but this may be related to the relatively low number of clones sequenced. Dewar et al. [22] used pyrosequencing of the V2-V3 region of the 16S rRNA gene to analyze the microbiota of four species of penguins: King (number of individuals not specified), Gentoo (four birds), Macaroni (four), and Little (four). The number of sequences obtained was respectively 132,340, 18,336, 6,324, and 4,826, and the corresponding numbers of OTUs were 1,331, 2,195, 1,362, and 561 (at 97% similarity, Table 3).

**Table 3:** Percentage of microbiota belonging to the main Phyla in different penguins and characteristics of the data sets used in each case.

Phylum	Little^a^	King^a^	Macaroni^a^	Gentoo^a^	Adélie^b^	Chinstrap^c^
**Bacterial composition**						
Firmicutes	24	47	43	18	39	60
Bacteroidetes	22	17	18	7	10	17.5
Proteobacteria	30	4	30	18	5	11
Fusobacteria	1	3		55		9
Actinobacteria	6		3	1	30	3.6
Planctomyces	11					
Spirochaeta			1			
Tenericutes	2					1.7
SR1		1		1		
TM7		1				
Other	1		1		17	
Unclassified	1	2	1			
**Data set characteristics**						
Individuals	4	?	4	4	6	53
Sequences	4,826	132,340	6,324	18,336	183	1,006,428
OTUs	561	1,331	1,362	2,195	52	3,621
% similarity of OTUs	97	97	97	97	99	97
Shannon index	2.5	2.9	3.3	2.4	2.5–2.8	2.97

^a^ Dewar et al. (2013).

^b^ Banks et al. (2009).

^c^ Present work.

In the present study, we analyzed the composition of the GIT microbiota of the Chinstrap penguin considering a much larger number of individuals (53 in total) and an order of magnitude larger number of sequences (around one million) than the previous studies, thus providing a robust data set for future reference. We retrieved a total of 3,621 OTUs at 97% similarity. The absolute numbers of OTUs cannot be easily compared among studies because of the very different number of sequences, particularly low in the Adélie study. However, we can assume that the composition and diversity indices will reflect the most abundant members of the microbiota and, therefore, can be compared. The Shannon index varied between 2.4 in Gentoo and 3.3 in Macaroni (Table 3), a relatively small range, suggesting there are no major differences in diversity among the different penguin species. These values are much lower than those found in poultry (between 5 and 6) also using pyrosequencing and a 97% similarity level [38]. The latter study analyzed over 600,000 sequences and was therefore comparable to ours in absolute numbers. These authors using the Chao estimator calculated between 200 and 400 OTUs per sample. This fits very well with our average Chao of 348 for adults (Table 1). Therefore, the microbiota of the chicken has similar OTU richness than that of Chinstrap penguins, but a much larger diversity. This indicates that the microbiota of chickens presents less dominance and higher evenness. Possible reasons for this are intriguing, but probably the artificial complex diets of broiler chickens and the more specialized diets of wild penguins may have influenced results.

In fact, diet has been shown to be a powerful predictor of GIT microbiota composition in mammals [39]. The gut microbiota of herbivorous monkeys was closer to that of other herbivorous mammals such as horses or cows, than to that of human beings, whose microbiota is closer to that of carnivores than to those of herbivores. One characteristic of the carnivore microbiota was the small representation of Bacteoridetes, as opposed to their importance in herbivores and omnivores [39]. This kind of correlation is less clear when birds are considered alone [5]. Gulls, chickens, and parrots have only 1.1, 1.9, and 0.2% of Bacteroidetes. Gulls can perhaps be considered as carnivores. However, chickens and parrots are omnivores, while other omnivores such as ostriches and turkeys have large percentages of Bacteroidetes (see review in [5]).

In the case of penguins, Bacteroidetes ranged between 7% in Gentoo and 22% in Little. There were clear differences among the three pygoscelids, with Adélie and Chinstrap presenting 10 and 17.5% respectively. The three species were also very different in overall composition (Table 3). Firmicutes was the most abundant phylum in Chinstrap while Fusobacteria accounted for 55% in Gentoo. In Adélie, Firmicutes (39%) and Actinobacteria (30%) were co-dominant. The latter, were only 1 or 3.6% in Gentoo and Chinstrap respectively. These major differences seem surprising for congeneric species, especially considering that their diets are relatively similar. It is true that Gentoos have a larger percentage of fish in their diet that the other two species (between 0.2 and 70% by weight, [40]), but krill represents most of the food for the three of them. Moreover, the composition of the two food items is very similar in fat (around 1.3%) and protein content (around 15%). The main difference between fish and krill seems to be the chitin exoskeleton of krill. Chitin is claimed to be degraded mainly by Bacteroidetes in aquatic environments and by Actinobacteria in soil environments [41], but the capability to degrade chitin is very extended among marine bacteria (see for example [42]). A diet rich in chitin such as that of *Pygoscelis* penguins should encourage dominance by chitin degraders. A metagenomic analysis of the gut microbiota of these penguins would be needed to determine the importance of chitin in the bacterial composition, as well as the presence of genes coding for proteolytic enzymes that could vary between penguin species according to their diet. Another factor that could drive a higher persistence of some bacterial groups is their capacity to detoxify certain compounds, like domoic acid acquired from fish [43] or fluoride from krill [44].

Age is another factor that can be important. We showed how composition changed substantially between chicks and adults. In particular, Bacteroidetes were much more abundant in adults than in chicks (Fig 2). Adults had a richer and more diverse GIT microbiota than chicks supporting findings of previous works with Tree Swallows [12] and poultry [45,46]. Given that chicks are fed exclusively on food regurgitated by adults, their decreased microbial diversity could be related to the lower digestion capabilities required, since food is partly predigested by the parents. In mammals including humans, gut microbiota changes with the introduction of solid food and becomes adult-like after one year of age [47]. Similarly, it is expected that penguins enrich their gut microbiome as their diet becomes richer and self-supplied. We found more OTUs in adults than in chicks which agrees with the results obtained by van Dongen et al. [11] in Black-legged Kittiwakes (*Rissa tridactyla*), but differs from findings by González-Braojos [48] in the Pied Flycatcher (*Ficedula hypoleuca*) who did not find an increase in the number of OTUs in nestlings from 7 to 13 days old. Such differences could be explained due to the large ecological differences (habitat, diet, nesting, breeding duration, life span etc.) between penguins and Black-legged Kittiwakes, both seabirds on the one hand, and the passerine Pied Flycatcher, whose nestlings are very likely to acquire the GIT bacteria at a very early age, on the other [48].

Shannon diversity index showed higher values for adults than chicks of Chinstrap Penguin. A similar result of age-dependent diversity was obtained by González-Braojos [48] with older nestlings showing a higher diversity than younger ones. Several factors shape bacterial colonization of GIT such as diet, environment, body condition, immune response, among others. Immune system has in fact been considered the strongest environmental pressure shaping GIT microbiota in animals [49]. The potential role of regurgitated food in the development of birds’ immune system and GIT microbiota needs to be elucidated.

Other determinants of microbiota composition are related to life history of the birds. Thus, Dewar et al. [50] showed that the microbiota of King and Little penguins changed during the period of fasting associated to moult. On the other hand, we found that nest influenced the GIT microbiota composition in chicks although this was not the case in adults. The difference between chicks and adults is consistent with the nest being the only environment for chicks, while adults spend most of their time elsewhere, reducing the potential influence of nest on GIT microbiota.

The fact that microbial composition was more similar among siblings than among non-relative chicks and their parents suggests an important role for environmental sources in GIT bacterial colonization. Genetic factors can also play a role in determining the bacterial assemblages as have been shown in other bird species [8,51]. However, our results showed that GIT microbiota similitude between chicks and their parents did not differ from the one found between chicks and non-related adults. This suggests that environmental factors are more influential than genetic ones on the composition of GIT in chinstrap penguin chicks. Nevertheless, the relative importance of both factors should be tested through cross-fostering experiments (see [8]).

In summary, the present work provides the most extensive analysis of the GIT microbiota of a penguin. This should provide a robust baseline for future studies of this and other species. The main phyla were the same as previously found for other penguin species ([21,22]). However, there were very large differences in composition even between the three members of the genus *Pygoscelis*. We also showed that age was a major determinant of microbiota composition, while nests had a significant influence only in chicks and suggest a higher importance of environmental over genetic factors in the composition of the microbiota of chicks.

## Supporting Information

S1 Table The most abundant genera in the cloacal microbiota of Chinstrap penguins. (PDF)

S2 Table Taxa responsible for the largest contribution (in %) to the dissimilarity between adults and chicks according to SIMPER analysis. (PDF)
