# Supplementary material for: Age-Related Differences in the Gastrointestinal Microbiota of Chinstrap Penguins (Pygoscelis antarctica)
Source: PLoS One. 2016 Apr 7;11(4):e0153215. doi: 10.1371/journal.pone.0153215 (PMC4824521; doi:10.1371/journal.pone.0153215)
Supplement: S1 Table — (PDF) [file pone.0153215.s001.pdf]

S1 Table. The most abundant genera in the cloacal microbiota of Chinstrap penguins.

Phylum	*Genus*	Reads
**Firmicutes**	Incertae,Sedis	284278
	Alkaliphilus	44044
	Anaerotruncus	37049
	Clostridium	31089
	Finegoldia	20651
	Leuconostoc	20602
	Fastidiosipila	13524
	Peptoniphilus	12057
	Peptoniphilus	12057
	Erysipelothrix	11559
	Sporosarcina	9288
	Gallicola	7681
	Dialister	7025
	Tissierella	5836
	Lactococcus	4335
	Helcococcus	3297
	Candidatus, Arthromitus	2931
	Filifactor	2797
	Negativicoccus	2724
	Guggenheimella	2361
	Sarcina	2044
	Staphylococcus	2025
	Peptococcus	1799
	Tepidimicrobium	1623
	Carnobacterium	1425
	Murdochiella	1030
**Fusobacteria**	*Fusobacterium*	65457
	Cetobacterium	25659
**Bacteroidetes**	*Flavobacterium*	82660
	Petrimonas	65457
	Porphyromonas	26435
	Proteiniphilum	7441
	Blvii28,wastewater-sludge,group	7241
	Dysgonomonas	5998
	Ornithobacterium	4369
	Paludibacter	4276
	Gelidibacter	1481
**Proteobacteria**	*Campylobacter*	14932
	Chelonobacter	14001
	Snodgrassella	11600
	Escherichia-Shigella	3828
	Pseudomonas	3227
	Psychrobacter	2609
	Helicobacter	1443
	Sutterella	1263

	Cardiobacterium	1071
**Actinobacteria**	*Corynebacterium*	11848
	Actinomyces	10853
	Georgenia	1744
