# Supplementary material for: Age-Related Differences in the Gastrointestinal Microbiota of Chinstrap Penguins (Pygoscelis antarctica)
Source: PLoS One. 2016 Apr 7;11(4):e0153215. doi: 10.1371/journal.pone.0153215 (PMC4824521; doi:10.1371/journal.pone.0153215)
Supplement: S2 Table — (PDF) [file pone.0153215.s002.pdf]

S2 Table. Taxa responsible for the largest contribution (in %) to the dissimilarity between adults and chicks according to SIMPER analysis. Blue indicates OTUs that were more abundant in chicks and green in adults.

			**Average abundance**		**Contribution**	**Taxonomy**			
**OTU**	**Reads**	**Samples[a]**	**Adults**	**Chicks**	**%**				
3116	156701	45	4.73	64.20	2.99	Clostridiales	Peptostreptococcaceae	Incertae.Sedis	uncultured.bacterium
1114	61449	44	5.45	33.70	1.4	Fusobacteriales	Fusobacteriaceae	Fusobacterium	Fusobacterium,sp.,RMA,1065
3567	29107	24	0.40	24.17	1.15	Clostridiales	Lachnospiraceae	Incertae,Sedis	uncultured,bacterium
258	35112	16	0.35	16.24	0.75	Clostridiales	Ruminococcaceae	Anaerotruncus	uncultured,bacterium
3709	10585	23	0.33	15.68	0.75	Clostridiales	Lachnospiraceae	Incertae,Sedis	Clostridium,colinum
243	14001	45	6.02	14.99	0.66	Clostridiales	Ruminococcaceae	Fastidiosipila	Clostridiales,genom.,BVAB3,str.,UPII9-5
2605	19763	34	11.95	12.94	0.71	Lactobacillales	Leuconostocaceae	Leuconostoc	uncultured,bacterium
3720	18388	14	0.22	12.70	0.62	Clostridiales	Lachnospiraceae	Incertae,Sedis	Clostridium,colinum
947	36881	42	26.50	10.71	1.15	Clostridiales	Family,XI,Incert Sedis	Incertae,Sedis	uncultured,bacterium
3457	50361	35	30.18	8.78	1.49	Bacteroidales	Porphyromonadaceae	Petrimonas	uncultured,bacterium
3276	24361	39	9.48	7.15	0.66	Fusobacteriales	Fusobacteriaceae	Cetobacterium	uncultured,bacterium
2004	25420	34	25.95	5.33	1.17	Bacteroidales	Porphyromonadaceae	Petrimonas	uncultured,bacterium
1248	43765	34	32.86	5.06	1.44	Clostridiales	Clostridiaceae	Alkaliphilus	uncultured,bacterium
2547	12019	29	15.64	2.10	0.78	Bacteroidales	Porphyromonadaceae	Porphyromonas	Porphyromonas,sp..2018
3587	9236	32	14.95	2.03	0.67	Bacteroidales	Porphyromonadaceae	Porphyromonas	Porphyromonas,gingivalis,ATCC,33277
734	14525	32	20.68	1.85	0.91	Campylobacterales	Campylobacteraceae	Campylobacter	uncultured,bacterium
2964	11442	29	16.73	0.23	0.79	Neisseriales	Neisseriaceae	Snodgrassella	Snodgrassella,alvi
2425	10262	28	16.66	0.22	0.77	Neisseriales		SC-I-84	uncultured,proteobacterium
2467	19988	27	22.31	0.21	1.06	Clostridiales	Family,XI,Incert Sedis	Finegoldia	uncultured,bacterium
2858	5878	27	12.60	0.09	0.58	Candidate,div SR1			uncultured,bacterium

[a] Number of samples in which the OTU appeared.
